# Prognostic Importance of Axillary Lymph Node Response to Neoadjuvant Systemic Therapy on Axillary Surgery in Breast Cancer—A Single Center Experience

**DOI:** 10.3390/cancers16071306

**Published:** 2024-03-27

**Authors:** Cvetka Grašič Kuhar, James Geiger, Fabienne Dominique Schwab, Viola Heinzelmann-Schwartz, Marcus Vetter, Walter Paul Weber, Christian Kurzeder

**Affiliations:** 1 Department of Medical Oncology, Institute of Oncology, 1000 Ljubljana, Slovenia; 2 Faculty of Medicine Ljubljana, University of Ljubljana, 1000 Ljubljana, Slovenia; 3 Breast Cancer Center, University Hospital Basel, University of Basel, 4001 Basel, Switzerland; james.geiger@usb.ch (J.G.); fabienne.schwab@usb.ch (F.D.S.); walter.weber@usb.ch (W.P.W.); christian.kurzeder@usb.ch (C.K.); 4 Department of Gynecologic Oncology, University Hospital Basel, 4031 Basel, Switzerland; viola.heinzelmann@usb.ch; 5 Medical Faculty, University of Basel, 4001 Basel, Switzerland; marcus.vetter@ksbl.ch; 6 Department of Hematology and Oncology, Cantonal Hospital Basel-Land, 4410 Liestal, Switzerland

**Keywords:** breast cancer, neoadjuvant systemic therapy, pathologic complete response, nodal response, axillary surgery

## Abstract

**Simple Summary:**

Neoadjuvant treatment refers to therapy given before surgery for early-stage breast cancer. It has several benefits, especially for patients with specific subtypes like HER2+ and triple-negative cancers. By using neoadjuvant treatment, tumors can shrink before surgery, allowing for less invasive surgical procedures in the breast and axilla. Achieving complete tumor disappearance after neoadjuvant treatment is associated with better survival outcomes. Researchers studied 92 patients initially diagnosed with node-positive disease. A key finding is that patients with HER2+ and triple-negative subtypes who have achieved complete tumor disappearance in the breast have a higher chance of achieving complete tumor disappearance in the axilla, which allows for less aggressive axillary surgery. In this study, more than half the patients did not need a removal of all the nodes in the axilla. In summary, neoadjuvant treatment improves outcomes, reduces surgical morbidity, and benefits patients with HER2+ and triple-negative subtypes.

**Abstract:**

Neoadjuvant systemic treatment (NST) is the standard treatment for HER2+, triple-negative (TN), and highly proliferative luminal HER2− early breast cancer. Pathologic complete response (pCR) after NST is associated with improved outcomes. We evaluated the predictive factors for axillary-pCR (AXpCR) and its impact on the extent of axillary node surgery. This retrospective study included 92 patients (median age of 50.4 years) with an initially node-positive disease. Patients were treated with molecular subtype-specific NST (4.3% were luminal A-like, 28.3% luminal HER2−, 26.1% luminal HER2+, 18.5% HER2+ non-luminal, and 22.8% TN). Axillary-, breast- and total-pCR were achieved in 52.2%, 48.9%, and 38% of patients, respectively. In a binary logistic regression model for the whole population, the only independent factor significantly associated with AXpCR was breast-pCR (OR 7.4; 95% CI 2.6–20.9; *p* < 0.001). In patients who achieved breast-pCR, aggressive subtypes (HER2+ and TN; OR 11.24) and clinical tumor stage (OR 0.10) had a significant impact on achieving AXpCR. Axillary lymph node dissection was avoided in 53.3% of patients. In conclusion, in node-positive patients with HER2+ and TN subtypes, who achieved breast-pCR after NST, de-escalation of axillary surgery could be considered in most cases.

## 1. Introduction

A significant advancement in the management of patients with node-positive early-stage breast cancer is the widespread adoption of neoadjuvant systemic therapy (NST). Response to therapy allows for a possible de-escalation of surgical intervention in the axilla and constitutes an important predictive factor for adjuvant treatment escalation according to breast cancer molecular subtypes defined by hormonal receptor status and HER2 overexpression. 

In one of the earliest studies on NST including patients with clinical node-positive disease (cN+), axillary pathologic complete response (AXpCR) was achieved in 22% of the patients [1]. Total-pCR in response to NST, defined as no invasive cancer in the breast and axillary nodes (ypT0/Tis ypN0), was reported in only 13% of the patients, and was the highest in the HER2-positive (33%) and triple-negative (TN) subtypes (30%) [2]. Over the last decade, the proportion of patients that achieved pCR in response to NST substantially increased, particularly in the HER2-positive and TN subtypes [3].

Traditionally, the standard surgical procedure for cN+ axilla after NST is axillary lymph node dissection (ALND). This procedure, however, is linked with substantial risks of arm-related symptoms like chronic pain, lymphoedema, loss of skin sensitivity, and missense feelings, thus diminishing the quality of life of patients. With a higher proportion of AXpCR, we are witnessing a trend towards less aggressive axillary surgeries, such as sentinel lymph node biopsy (SLNB) [4,5,6,7,8], SLNB in combination with the removal of a clipped axillary lymph node, and targeted axillary dissection [5,9]. As a radiological assessment of the axillary response after NST does not accurately predict AxpCR [10], additional prognostic factors are needed to identify the patients in which ALND could be safely avoided.

We performed a retrospective observational study in patients with cN+ disease treated with NST at a single cancer center. The first aim of this study was to determine the treatment outcomes according to the molecular subtypes, evaluated as AXpCR, breast-pCR and total-pCR. The second aim was to evaluate the predictive factors for AXpCR in patients with node-positive breast cancer and its impact on axillary surgery and relapse-free survival (RFS).

## 2. Materials and Methods

### 2.1. Patients and Treatment 

Patients with node-positive early-stage breast cancer, diagnosed and treated at the University Hospital Basel between November 2012 and August 2020, were included in the study. The study inclusion criteria were cN1-3 (by ultrasound and/or PET CT), cT1-4, and to be fit for NST. The exclusion criteria were cN0, metastatic disease, or refusing or being unfit to receive NST. All patients had a core biopsy of the primary tumor, ultrasound of the axilla with biopsy-proven nodal involvement, insertion of a marking clip in the breast and axillary node, and a staging for distant metastases (PET CT or CT scan of neck, thorax and abdomen, and whole-body bone scintigraphy). The definition of molecular subtypes was based on a pathohistological report on the core biopsy, which included the expression of estrogen (ER) and progesterone receptors (PR), and HER2. ER and PR were positive in the case of ≥1% expression. HER2 was positive when there was an immunohistochemical staining of 3+ or in the case of 2+, by a HER2 to chromosome 17 (HER2:CEP17) ratio of ≥2.0 analyzed by dual-probe in situ hybridization (according to ASCO–CAP guidelines) [11]. Ki-67 ≥ 20% was assigned as high. Molecular subtypes were defined as recommended by the St. Gallen Consensus [12]. Luminal A-like subtype was defined as high ER and PR, low Ki-67 (<20%), and low tumor burden. Luminal HER2-negative was defined as ER or PR ≥ 1%, Ki-67 ≥ 20% and HER2-negative. Luminal HER2-positive was defined as HER2-positive and ER or PR ≥ 1%. HER2-positive (non-luminal) was defined as HER2-positive, ER = 0%, and PR = 0%. A subtype was classified as triple-negative (TN), if ER = 0%, PR = 0%, and HER2-negative.

Treatment consisted of NST (chemotherapy ± anti-HER2 agents or immune checkpoint inhibitors, depending on the subtype and year of treatment), followed by breast surgery (mastectomy or breast-conserving surgery) and surgery of the axilla (SLNB, SLNB + targeted axillary surgery (TAS), or ALND). TAS was defined by the palpation-guided selective removal of presumed nodal disease in combination with the SLNB procedure, and the targeted resection of the nodes which were biopsy proven and clip marked before NST. Breast MRI and ultrasound of the axilla were performed before surgery.

### 2.2. Endpoints

Primary endpoints were AXpCR, breast-pCR, and total-pCR according to the molecular subtypes. AXpCR was defined as no micro- or macro-metastases in axillary lymph nodes after NST (ypN0). Breast-pCR was defined as the absence of invasive carcinoma with possible residual tumor in situ (ypT0/Tis). Total-pCR was regarded as no residual invasive tumor cells in either the breast or axillary nodes (ypT0/Tis ypN0). 

The secondary endpoint was to evaluate the association of AXpCR with clinical, pathological, and treatment factors. The impact of AXpCR on the types of axillary surgery and RFS was analyzed. RFS was determined as the time elapsed between the date of diagnosis to the date of the first evidence of locoregional invasive recurrence, distant metastasis, or death.

### 2.3. Statistical Analysis

Demographic information of the patients, tumor characteristics, and treatment specifics were presented using frequency distributions and proportions for categorical data, while age was described using the median and range. Statistical analyses involved the use of Pearson’s chi-squared test for categorical variables and an unpaired Student’s *t*-test for comparing age across different groups. In cases where the expected values were less than 5% in over 20% of cells, Fisher’s Exact test was utilized, allowing for analysis with smaller sample sizes. A significance level of *p* ≤ 0.05 was considered statistically significant. Binary logistic regression was performed to explore the possible associations of clinical, pathological, and treatment regimens with AXpCR, and reported these as an odds ratio (OR) with 95% confidence intervals (CI). For an estimation of the median follow-up time, the reverse Kaplan–Meier method was used. The estimated survival curve for RFS was generated by the Kaplan–Meier method and compared using the log-rank test. The prognostic significance was expressed by a hazard ratio (HR) and 95% CI. All statistical analyses were performed using SPSS v.26 (IBM Corp., Armonk, NY, USA).

## 3. Results

### 3.1. Patient Population

Our study population consisted of 92 women with histologically proven node-positive early-stage breast cancer. The patients’ clinicopathological characteristics are presented in Table 1. The median age of the patients was 50.6 (range: 27–88) years. According to the subtypes, 4.3% were luminal A-like, 28.3% luminal HER2-negative, 26.1% luminal HER2-positive, 18.5% HER2-positive non-luminal, and 22.8% TN. Patients of different subtypes had no statistically significant differences in the following clinicopathological characteristics: age, cT stage, cN stage, grade, and body mass index. Half of the patients presented as cT1 (53.3%), and 75% as cN1.

### 3.2. Treatment

Treatments with NST (chemotherapy, anti-HER2 therapy, immune checkpoint therapy), surgery, and adjuvant therapy are presented in Table 2. 

NST was significantly different among the molecular subtypes. Sequential administration of dose-dense anthracyclines and weekly taxanes was the most used chemotherapy regimen (in 42.4% of all patients); it was applied in 80.9% of the luminal HER2-negative subtype, and in one-third of patients having HER2-positive (luminal and non-luminal) subtype. Taxane plus platinum (docetaxel + carboplatin) were provided in half of the HER2-positive tumors. In TN, 71% received a sequential regimen of dose-dense anthracyclines and taxanes + carboplatin, or taxanes + carboplatin alone (14%). The median number of chemotherapy cycles was eight (range: 0–10). Patients with the HER2-positive subtypes were treated concurrently with anti-HER2 targeted therapy and taxanes. In the neoadjuvant setting, 83% and 71% of HER2-positive patients (luminal and non-luminal, respectively) were treated by a dual HER2 blockade consisting of trastuzumab and pertuzumab, while the others received only trastuzumab (except for one patient with breast cancer during pregnancy). All the HER2-positive patients received adjuvant anti-HER2 therapy; a half trastuzumab, others either a dual blockade or trastuzumab–emtansine. Nineteen percent of the TN patients received neo- and adjuvant immune checkpoint inhibitor therapy with pembrolizumab. Adjuvant chemotherapy was delivered to 8.7% of patients. All the patients with the luminal A and luminal HER2-negative subtypes, and 96% of the HER2-positive luminal subtype, were treated with adjuvant endocrine therapy (80% with aromatase inhibitors and 20% with tamoxifen). 

### 3.3. pCR According to Molecular Subtypes

**Figure 1:**
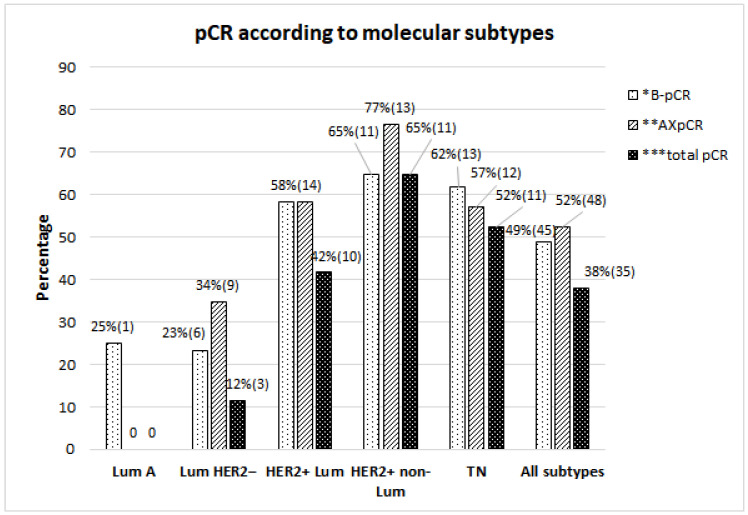
Pathologic complete response (pCR) in the breast, axilla, and both the breast and axilla (total-pCR) according to different molecular subtypes. * *p* = 0.029, ** *p* = 0.019, *** *p* = 0.002. Values are expressed in percentages and absolute values in parenthesis. Lum: luminal, TN: triple-negative.

Following the NST, 48 patients (52.2%) achieved AXpCR, higher than breast-pCR (45 patients; 48.9%). Total-pCR was present in 35 patients (38%). Percentages of AXpCR, breast-pCR and total-pCR were significantly different across the subtypes (Figure 1). The highest AXpCR (76.5%) was achieved in the HER2-positive non-luminal subtype, followed closely by the TN and HER2-positive luminal subtypes.

### 3.4. Association of Clinical, Pathological, and Treatment Factors with AXpCR

A comparison of the characteristics between patients who achieved AXpCR and those who did not are presented in Table 3. Molecular subtype (*p* = 0.013), anti-HER2 treatment (*p* = 0.031), and breast-pCR (*p* < 0.0001) were significantly associated with AXpCR in a univariate analysis. Initial tumor and nodal stages were not associated with AXpCR. A binary logistic regression model for the whole population showed that only breast-pCR was significantly associated with AXpCR. Patients who achieved breast-pCR had 7.4 times higher odds of having AXpCR than patients without breast-pCR (OR 7.42; 95% CI 2.64–20.89; *p* < 0.001).

**Figure 2:**
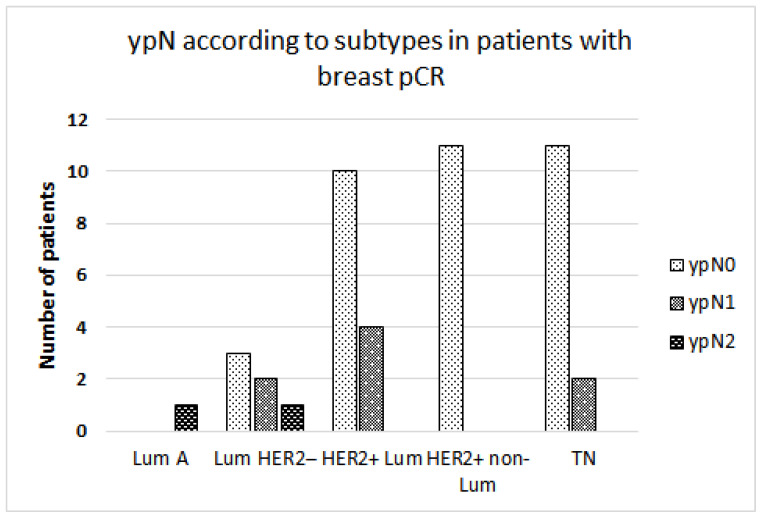
The pathologic nodal stage following neoadjuvant systemic treatment is presented according to molecular subtypes. A subgroup of patients having a pathologic complete response in the breast is presented. pCR: pathologic complete response. TN: triple-negative; Lum: luminal.

We further analyzed a group of patients with breast-pCR. Among the patients who achieved breast-pCR, molecular subtype (HER2-positive and TN) and clinical T stage (but not cN stage) correlated with AXpCR (Table 4, binary logistic regression). Axillary residual disease in the patients who achieved breast-pCR is presented in Figure 2. Patients with the HER2-positive non-luminal subtype, who achieved breast-pCR, all achieved AXpCR as well. In the HER2-positive non-luminal and TN subtypes, residual axillary disease burden was low (mostly no residual disease (ypN0) or ypN1) thus allowing the de-escalation of axillary surgery.

### 3.5. Surgical Treatment

Regarding breast surgery, 56.5% of all patients were treated with breast-conserving surgery and 43.5% with a mastectomy.

**Figure 3:**
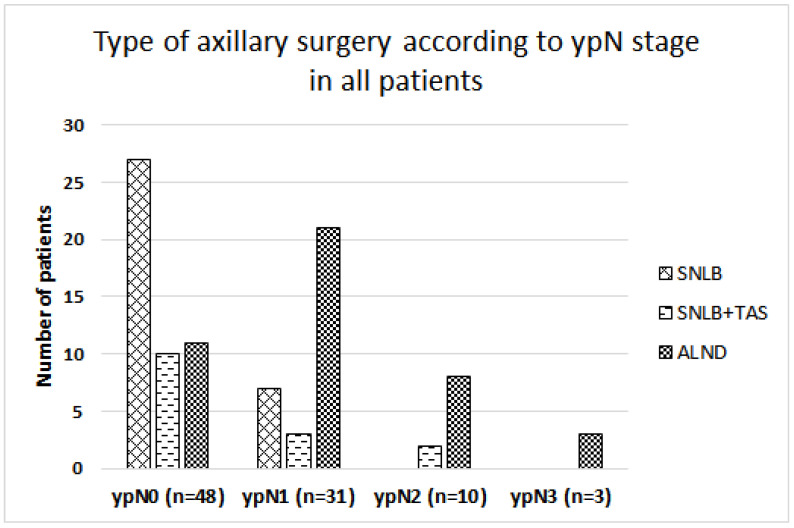
Axillary surgery after neoadjuvant systemic therapy stratified by the pathologic nodal stage. SLNB: sentinel lymph node biopsy; TAS: targeted axillary surgery; ALND: axillary lymph node dissection.

Management of the axilla was as follows: SLNB (37%), SLNB + TAS (16.3%), and ALND (46.7%). The mean number of extirpated lymph nodes was 3.5 (range: 2–6), 5.6 (range: 3–8), and 17 (range: 8–31) in SLNB, SLNB + TAS, and ALND procedures, respectively. In summary, 53.3% of patients were spared ALND. Axillary surgery significantly depended on the residual nodal stage (ypN stage) (Figure 3; *p* < 0.001). Shortly, in the case of achieving AXpCR, patients had less extensive axillary surgery. Still, 11 patients with AXpCR had unnecessary ALND. In the case of breast-pCR, only 14/45 patients had ALND compared to 29/47 without breast-pCR. There were very few patients with ypN2 and ypN3 (10 and 3 patients, respectively). Of them, 12 had ALND, 1 had SLNB and TAS (stage ypN2); all but 1 had adjuvant RT. In the ypN1 group (*n* = 31) 23.5% had SLNB plus breast RT, 9.7% had SLNB and TAS plus RT (1 of 3 patients refused RT), and 70% ALND (92.7% with RT).

### 3.6. Adjuvant Radiation Therapy

**Figure 4:**
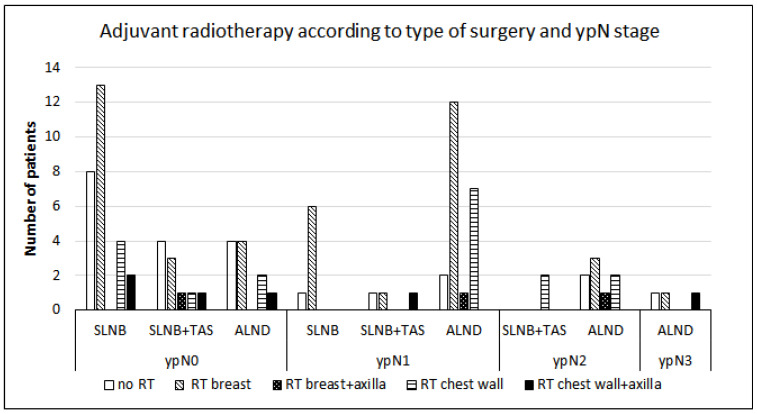
Adjuvant radiotherapy according to type of surgery and residual nodal stage (ypN stage) after neoadjuvant systemic treatment. SLNB: sentinel lymph node biopsy; TAS: targeted axillary surgery; ALND: axillary lymph node dissection; RT: radiotherapy.

Sixty-nine patients (75%) received adjuvant radiation therapy (RT). HER2-positive non-luminal and TN subtypes had RT less often than other subtypes (*p* = 0.002). Four patients (4.3%) refused RT. Breast and regional nodal irradiation were performed in 45 patients (48.9%), and chest wall irradiation in 24 patients (27.1%). In eight patients (two in the breast RT group and six in the chest RT group), the axilla was included in the radiation field. RT according to the type of surgery and ypN stage is presented in Figure 4.

### 3.7. Relapse-Free Survival

The median follow-up time was 2.9 years. Thirteen patients (14.1%) relapsed and three (3.3%) died. The sites of relapse were as follows: 1 local, 1 locoregional, 1 locoregional + distant, and 10 distant relapses (of the later, 1 had leptomeningeal and 2 had brain metastases). Median RFS was not reached. One- and two-year RFS were 97.6% and 88.5%, respectively. RFS depended on AXpCR and breast-pCR. Patients achieving AXpCR and breast pCR had significantly higher RFS compared to their counterparts (*p* = 0.011 and 0.042, respectively). Cox proportional hazard analysis revealed AXpCR as the only prognostic factor for RFS in the univariate analysis (HR 0.18; 95% CI 0.04–0.80; *p* = 0.024). Other factors (type of breast and axillary surgery, adjuvant RT, adjuvant endocrine therapy, adjuvant anti-HER2 therapy, molecular subtype, breast-pCR and total-pCR) were not statistically significant in our sample (Table 5); however, our sample size was small, and the median follow-up time was short.

## 4. Discussion

The potential of NST to reduce the extent of breast surgery is well established; however, its potential to reduce axillary surgery is less well evaluated. As almost half of the patients could achieve AXpCR with NST, there is an unmet need to find prognostic/predictive factors for it to de-escalate axillary surgery and avoid arm-related symptoms. In our study on cN+ early-stage breast cancer patients treated with NST, the percentage of AXpCR was 52.2%. Notably, the achievement of AXpCR was highly associated with breast-pCR. In patients who achieved breast-pCR, AXpCR was higher in aggressive subtypes (HER2-positive and TN) and in lower clinical T stage. This allowed us to perform less extensive axillar surgery (SLNB or SNLB +TAS). As a result, 53.3% of patients avoided ALND.

Adjuvant chemotherapy was given in 8.7% of our patients. The paradigm of adjuvant (i.e., post-neoadjuvant) treatment started after the publication of the positive results of the CREATE-X study for the TN subtype (2017) and Katherine for the HER2-positive subtype (2019) [13,14]. As our study includes patients from 2012 onwards, only a small proportion of the non-pCR patients received this treatment.

Several studies evaluated the association of AXpCR with a clinical node status before NST. The rate of involved nodes after NST was 2–22% in cN0 patients [15,16,17], 34–59% in cN1 patients [5,17], and 20–61%, depending on subtype, in cN+ patients [18,19]. In real-world clinical practice, patient outcomes often tend to be worse compared to those observed in prospective studies, or they achieve comparable results but with a delay. However, we promptly incorporated new scientific data into routine clinical practice. In our retrospective study, more than half of the patients with HER2-positive and TN subtypes achieved AXpCR and breast-pCR (Figure 1). The highest AXpCR (76.5%), breast-pCR (64.7%), and total-pCR (54.7%) were observed in the HER2-positive non-luminal subtype. These results are in line with the results in a randomized study, TRYPHAENA, with 64.6% achieving total-pCR [20]. Similarly, in the TN subtype with 57.1% AXpCR and 52.4% total-pCR, our results are comparable with recently published results on NST with chemotherapy and the addition of immune checkpoint inhibitors. In the pembrolizumab arm, total-pCR increased from 51.2% to 64.5% [21], and from 41% to 58% in the atezolizumab arm [22], when compared to the chemotherapy only arm.

The most important finding of our study was that AXpCR is highly associated with breast pCR (in HER2-positive non-luminal patients in 100%) (Table 3). Association of AXpCR with breast-pCR was also recently reported by other authors [1,8,15,16,17,23,24,25]. Additional factors associated with AXpCR in the literature include molecular subtype, cT stage [24,26], age [26], cN stage [17], molecular subtype (but not cN stage) [27], estrogen receptor negativity, and high-grade, clinical complete response in the breast and axillary nodes [19]. Barron et al. found that breast-pCR in HER2−positive and TN disease was highly correlated with AXpCR in cN0 patients only [15]. Contrary to this finding, we demonstrated an association of AXpCR with HER2−positive and TN subtypes in cN+ patients; however, only in patients who achieved breast-pCR. In addition, cT stage (but not cN stage) was associated with AXpCR.

The high AXpCR rate within our study implicates the possibility of de-escalating axillary surgery. Several prospective trials have shown that SLNB after NST is feasible; however, these trials included different patient populations. In the TransSENTINA trial, the AXpCR rate of patients with cN0 disease (who also had occult metastases on preoperative SLNB) was 78% [16]. In the Z1071 trial, the AXpCR rate of patients with cN1 stage was 41% [5]. The NSABP B-27 trial showed that SLNB after NST was not inferior to ALND in terms of disease-free and overall survival [6]. The false negative rates of detecting sentinel lymph nodes (SLN) in the NST trials were 8.4–14.2%, and the SLN identification rates were 80–93% [3,4,5,6,7,8,16]. The false negative rate was lower (4%) when ≥3 nodes were removed [28], or 2–4% in the combination of SLNB with targeted axillary dissection; in the latter, the SLN identification rate was 100% [29]. National Comprehensive Cancer Network guidelines recommend marking the biopsied nodes to document their removal (targeted axillary dissection) using dual tracers, and removing at least 3 SLN [30]. Barrio et al. reported that such a procedure in cN1 disease, that converts to ycN0, is acceptable since nodal recurrence is a rare event [31]. Many patients underwent adjuvant radiation therapy and long-term results showed equivalent long-term disease-free survival.

There are limited data on cN2 patients. In patients with >3 clinically positive nodes at diagnosis, before NST or ypN1, the Lucerne toolbox recommends ALND at surgery [32]. Garcia-Tejedor et al. recently reported 40% AxpCR in the cN2 population, and that HER2-positive and TN tumors had the highest AXpCR rates, which poses the possibility of SLNB even in this high-risk subpopulation [33].

On the other extreme in the management of the axilla after NST is the testing of complete omission of axillary surgery in cN0 patients with HER2-positive or TN disease in the ASICS (NCT04225858) and EUBREAST-01 trials [34]. In the EUBREAST-01 trial, patients with radiologic complete remission in the axilla and breast-pCR will forgo axillary surgery [34]. Similarly, the ASICS trial will test the non-inferiority of omitting axillary surgery in HER2-positive and TN patients based on the imaging response. Pfob et al. recently reported a trial with an intelligent vacuum-assisted biopsy to identify patients with total-pCR (ypT0/ypN0) after NST for omission of both breast and axillary surgery [35]. However, pathologic staging of breast and axilla is very important, and forgoing this procedure in exceptional responders needs closer observation of possible relapse [36].

A high rate of AXpCR in our study allowed us to perform less aggressive axillary surgery, and thus it probably caused lower probability for comorbidity of the upper extremity/axilla (i.e., pain, lymphoedema, disturbances in sensation). In our report, 36% of patients underwent SLNB. Of them, 75% initially had stage cN1 and 25% ≥cN2. In the Mayo clinic report focusing on patients with cN1 stage only, SLNB was possible in 22% [37]. More effective systemic therapy in recent years probably contributed to the shift toward less extensive surgery of the axilla. In a recent study, Tinterri et al. compared the outcome of patients with cN0 and cN+ converting into ycN0 after NST [38]. For the detection of SLNB, a single tracer (99Technetium-labeled radiocolloid) was used, and ALND was needed in only 29.4% of cases, very similar to our results (in ypN0 patients, 22.9% had ALND). After 3.5 years, axillary recurrences were low in the cN0 and cN+ groups (<3%), and no differences in DFS and OS were found [38].

Patients with limited residual nodal disease, micro-metastases, or 1–2 affected lymph node (classified as part of ypN1 stage) might be considered the next group eligible to de-escalate surgery. Namely, in the setting of upfront surgery, patients with 1–2 involved sentinel nodes could have only SLNB, and ALND could be safely avoided, if they are planned for treatment with adjuvant radiation therapy [39]. Analogous with this, Chen et al. raised the question whether patients who achieved breast-pCR could be predictive for those who might be eligible to avoid ALND [25]. But the omission of ALND in low volume residual axillary disease (ypN1) was associated with an inferior five-year overall survival, therefore it should not be used outside of clinical trials [40]. Future de-escalation strategies in ypN+ patients include implementing protocol-based regional nodal irradiation. The ongoing international TAXIS trial (NCT03513614) aims to ascertain whether TAS combined with regional nodal irradiation including the full axilla is oncologically non-inferior and linked to enhanced quality of life in comparison to ALND and regional nodal irradiation excluding the dissected axilla [41].

We want to stress some strengths of our study. Firstly, we reported on the treatment results of cN+ breast cancer patients, which included very up-to-date NST treatment regimens and resulted in high rates of AXpCR, which is the most important prognostic factor for the long-term outcome in HER2-positive and TN subtypes [42]. Secondly, high rates of AxpCR allowed us to implement the de-escalation of axillary surgery, in terms of SLNB or SNLB + TAS. With this procedure, we could avoid ALND in almost half of our patients, which would have been all the candidates for ALND a decade ago, and thus we probably managed to decrease their morbidity. Additionally, a high proportion of non-anthracycline regimens in HER2-positive subtypes could abrogate cardiac toxicity, and additionally contribute to better long-term quality of life of patients.

This study has some limitations. Firstly, this is a retrospective study with the biases connected with this kind of analysis. Secondly, our study sample is small, single centered, and the follow-up time is too short to reliably assess the impact of less aggressive axillary surgery on relapse rate. However, in the aggressive subtypes of breast cancer, like TN and HER2-positive, we expected earlier relapses, in the first 2–3 years following surgery, but this was not the case. Relapses in the hormone receptor positive subtypes occurred later. Third, some patients who were treated at our facility have discontinued follow-up care at our facility due to the COVID-19 pandemic, while some relapses may have been treated in other facilities/countries.

## 5. Conclusions

An analysis of the treatment of clinically node-positive breast cancer with NST showed high breast- and AXpCR rate, especially in the HER2-positive and TN subtypes. In patients with these subtypes, who achieved breast-pCR, de-escalation of axillary surgery could be considered.

## Figures and Tables

**Table 1:** Baseline clinicopathological characteristics of the patients, stratified according to molecular subtypes of breast cancer.

Characteristics	All Patients N (%) 92 (100)	Luminal A N (%) 4 (4.3)	LumHER2− N (%) 26 (28.3)	LumHER2+ N (%) 24 (26.1)	HER2+ NonLum N (%) 17 (18.5)	Triple Neg. N (%) 21 (22.8)	*p*-Value
Age (Years) Median Range (min, max)	50.6 (27.0–88.2)	67.6 (35.5–75.6)	46.6 (27.0–78.5)	50.9 (33.0–74.6)	55.5 (32.0–88.2)	46.6 (38.3–87.4)	0.428
Age groups *n* (%) 20–35 years 36–50 years 51–65 years >65 years	5 (5.4) 40 (43.5) 26 (28.3) 21 (22.8)	0 (0) 1 (25) 0 (0) 3 (75)	3 (11.5) 13 (50) 7 (26.9) 3 (11.6)	1 (4.2) 9 (37.5) 9 (37.5) 5 (20.8)	1 (5.9) 4 (23.5) 5 (29.4) 7 (41.2)	0 (0) 13 (61.9) 5 (23.8) 3 (14.3)	0.097
Tumor stage *n* (%) Unknown (cT0/x) cT1 cT2 cT3 cT4	2 (2.2) 16 (17.4) 49 (53.3) 18 (19.5) 7 (7.6)	0 (0) 1 (25) 1 (25) 1 (25) 1 (25)	1 (3.8) 4 (15.4) 13 (50) 6 (23.1) 2 (7.7)	1 (4.2) 1 (4.2) 16 (66.7) 4 (16.6) 2 (8.3)	0 (0) 3 (17.7) 8 (47.1) 4 (23.5) 2 (11.7)	0 (0) 7 (33.3) 11 (52.4) 3 (14.3) 0 (0)	0.589
Nodal stage *n* (%) cN1 cN2 cN3	69 (75) 16 (17.4) 7 (7.6)	3 (75) 1 (25) 0 (0)	16 (61.5) 5 (19.2) 5 (19.2)	19 (79.2) 4 (16.7) 1 (4.2)	13 (76.5) 3 (17.6) 1 (5.9)	18 (85.7) 3 (14.3) 0 (0)	0.407
Grade *n* (%) Grade 1 Grade 2 Grade 3 Unknown	2 (2.2) 23 (25) 48 (52.1) 19 (20.7)	0 (0) 3 (75) 0 (0) 1 (25)	1 (3.8) 7 (27) 15 (57.7) 3 (11.5)	0 (0) 6 (25) 13 (54.2) 5 (20.8)	0 (0) 3 (17.7) 9 (52.9) 5 (29.4)	1 (4.8) 4 (19) 11 (52.4) 5 (23.8)	0.551

cT: clinical tumor stage; cN: clinical nodal stage.

**Table 2:** Treatment with different modalities, stratified according to molecular subtypes.

Characteristics	All Patients N (%) 92 (100)	Luminal A N (%) 4 (4.3)	LumHER2− N (%) 26 (28.3)	LumHER2+ N (%) 24 (26.1)	HER2+ NonLum N (%) 17 (18.5)	Triple Neg. N (%) 21 (22.8)	*p*-Value
Neoadj. Cht No ^1^ A→Tax TCb A→TCb Tax Capecitabine A ^2^	1 (1.1) 39 (42.4) 24 (26.1) 20 (21.7) 4 (4.3) 3 (3.3) 1 (1.1)	0 (0) 2 (50) 0 (0) 1 (25) 1 (25) 0 (0) 0 (0)	0 (0) 21 (80.9) 1 (3.8) 3 (11.5) 0 (0) 1 (3.8) 0 (0)	0 (0) 9 (37.5) 12 (50) 1 (4.2) 0 (0) 2 (8.3) 0 (0)	1 (5.9) 5 (29.4) 8 (47.1) 0 (0) 2 (8.3) 0 (0) 1 (5.9)	0 (0) 2 (9.5) 3 (14.3) 15 (71.4) 1 (4.8) 0 (0) 0 (0)	<0.001
Neoadjuvant antiHER2 th. No T TP	52 (56.5) 8 (8.7) 32 (34.8)	4 (100) 0 (0) 0 (0)	26 (100) 0 (0) 0 (0)	0 (0) 4 (16.7) 20 (83.3)	1 (5.9) ^2^ 4 (23.5) 12 (70.6)	21 (100) 0 (0) 0 (0)	<0.001
Neo/adjuvant ICI No Yes	88 (95.7) 4 (4.3)	4 (100) 0 (0)	26 (100) 0 (0)	24 (100) 0 (0)	17 (100) 0 (0)	17 (81) 4 (19)	0.007
Surgery of breast Mastectomy BCS	40 (43.5) 52 (56.5)	3 (75) 1 (25)	9 (34.6) 17 (65.4)	8 (33.3) 16 (66.7)	9 (52.9) 8 (47.1)	11 (52.4) 10 (47.6)	0.314
Surgery of axilla SLNB SLNB + TAS ALND	33 (35.9) 16 (17.4) 43 (46.7)	1 (25) 1 (25) 2 (25)	7 (26.9) 5 (19.2) 14 (53.8)	7 (29.2) 4 (16.7) 13 (54.2)	10 (58.5) 3 (17.6) 4 (23.5)	8 (38.1) 3 (14.3) 10 (23.5)	0.604
Adjuvant RT No Yes	23 (23.9) 69 (75)	0 (0) 4 (100)	2 (7.7) 24 (92.3)	3 (12.5) 21 (87.5)	10 (58.8) 7 (41.2)	8 (38.1) 13 (61.9)	0.002
Adjuvant Cht No Yes	84 (91.3) 8 (8.7)	3 (75) 1 (25)	25 (96.2) 1 (3.8)	22 (91.7) 2 (8.3)	16 (94.1) 1 (5.9)	18 (85.7) 3 (14.3)	0.540
Adj. antiHER2 th. No T TP T-DM1	51 (55.4) 20 (21.7) 15 (16.3) 6 (6.5)	4 (100) 0 (0) 0 (0) 0 (0)	26 (100) 0 (0) 0 (0) 0 (0)	0 (0) 12 (50) 6 (25) 6 (25)	0 (0) 8 (47.1) 9 (52.9) 0 (0)	21 (0) 0 (0) 0 (0) 0 (0)	<0.001
Adjuvant ET No Tamoxifen AI	39 (42.4) 7 (7.6) 46 (50)	0 (0) 0 (0) 4 (100)	0 (0) 3 (11.5) 23 (88.5)	1 (4.2) 4 (16.7) 19 (79.1)	17 (100) 0 (0) 0 (0)	21 (100) 0 (0) 0 (0)	<0.001

Cht: chemotherapy; ICI: immune checkpoint inhibitor (pembrolizumab); BCS: breast-conserving surgery; SLNB: sentinel lymph node biopsy; TAS: targeted axillary surgery; ALND: axillary lymph node dissection; A: anthracycline chemotherapy; Tax: taxane chemotherapy; TCb: taxane + carboplatin; T: trastuzumab; TP: trastuzumab + pertuzumab; T-DM1: trastuzumab emtansine; ET: endocrine therapy; AI: aromatase inhibitor. ^1^ an elderly patient had anti-HER2 treatment only; ^2^ a patient with breast cancer during pregnancy.

**Table 3:** Analysis of the factors associated with pathologic complete response in the axilla (AXpCR) to neoadjuvant systemic therapy.

Characteristics	No AXpCR N (%)	AX-pCR N (%)	*p*-Value
Tumor stage cT1/T2 cT3/T4	28 (43.1) 14 (56)	37 (56.9) 10 (44)	0.271
Tumor grade Grade I Grade II Grade III Unknown	2 (100) 15 (65.2) 18 (37.5) 9 (47.4)	0 (0) 8 (34.5) 30 (62.5) 10 (52.6)	0.056
Nodal stage *n* cN1 cN2/3	35 (45.5) 9 (60)	42 (54.5) 6 (40)	0.302
Chemotherapy Polychemotherapy Single-agent	39 (47) 4 (50)	44 (53) 4 (50)	0.579
Anti-HER2 th. No Yes	30 (57.7) 14 (35)	22 (42.3) 26 (75)	0.031
Subtype Lum A Lum HER2− Lum HER2+ HER2+ non-Lum TN	4 (100) 17 (64.5) 10 (41.7) 4 (23.5) 9 (42.9)	0 (0) 9 (34.6) 14 (58.3) 13 (76.5) 12 (57.1)	0.013
Breast-pCR No Yes	13 (27.7) 35 (77.8)	34 (72.3) 10 (22.2)	<0.0001

TN: triple-negative subtype; Lum: luminal.

**Table 4:** Odds ratios (OR) of achieving axillary pathologic complete response in patients having a breast pathologic complete response (*n* = 45).

Variable	OR (95% CI)	*p*-Value
Clinical tumor stage cT3/T4 vs. cT1/T2	0.10 (0.015–0.83)	0.033
Molecular subtype HER2+ (Lum + non Lum)/TN vs. Lum A/Lum HER2−	11.24 (1.18–107.10)	0.036
Clinical nodal stage cN2/3 and cN1	0.23 (0.03–1.58)	0.146

CI: confidence interval, TN: triple-negative; Lum: luminal subtype.

**Table 5:** Relapse-free survival: univariate analysis of possible prognostic factors. BCS: breast-conserving surgery; SLNB: sentinel lymph node biopsy; TAS: targeted axillary surgery; ALND: axillary lymph node dissection; pCR: pathologic complete response; TN: triple-negative, Lum: luminal.

Variable	HR (95% CI)	*p*-Value
Type of breast surgery (mastectomy vs. BCS)	0.36 (0.10–1.30)	0.119
Type of axillary surgery		
ALND	1.00	0.290
SLNB vs. ALND	0.31 (0.07–1.40)	0.126
SLNB + TAS vs. ALND	0.55 (0.07–4.40)	0.576
Adjuvant radiation (yes vs. no)	0.76 (0.23–2.46)	0.641
Adjuvant endocrine therapy (yes vs. no)	0.56 (0.31–1.03)	0.063
Adjuvant antiHER2 therapy (yes vs. no)	0.61 (0.44–1.97)	0.735
Molecular subtype (HER2+/TN vs. Lum A/Lum HER2−)	1.50 (0.46–4.89)	0.505
pCR in breast (yes vs. no)	0.24 (0.05–1.08)	0.062
pCR in axilla (yes vs. no)	0.18 (0.04–0.80)	0.024
Total pCR (yes vs. no)	0.19 (0.03–1.47)	0.112

## Data Availability

Data are contained within the article.
